# Epidermal Growth Factor Receptor Tyrosine Kinase Inhibitors in Treatment of Metastatic Non-Small Cell Lung Cancer, with a Focus on Afatinib

**DOI:** 10.3389/fonc.2017.00097

**Published:** 2017-05-16

**Authors:** Sami Morin-Ben Abdallah, Vera Hirsh

**Affiliations:** ^1^Department of Oncology, McGill University Health Centre, Montreal, QC, Canada

**Keywords:** non-small cell lung cancer, epidermal growth factor receptor, tyrosine kinase inhibitor, afatinib, gefitinib, erlotinib

## Abstract

Somatic epidermal growth factor receptor (EGFR) mutations are present in around 50% of Asian patients and in 10–15% of Caucasian patients with metastatic non-small cell lung cancer (NSCLC) of adenocarcinoma histology. The first-generation EGFR-tyrosine kinase inhibitors (TKIs) gefitinib and erlotinib have demonstrated improved progression-free survival (PFS) and response rates but not overall survival (OS) benefit in randomized phase III trials when compared with platinum-doublet chemotherapy. All patients treated with EGFR-TKIs will eventually develop acquired resistance to these agents. Afatinib, an irreversible ErbB family blocker, has shown in two randomly controlled trials in patients with EGFR-activating mutations, a significant improvement in PFS and health-related quality of life when compared to platinum-based chemotherapy. Afatinib improved OS in patients with Del19 mutations. In patients having progressed on first-generation EGFR-TKIs, afatinib did lead to a clinical benefit. A randomly controlled trial showed that PFS was significantly superior with afatinib vs. erlotinib in patients with squamous NSCLC in the second-line setting. A phase IIb trial comparing afatinib and gefitinib in first-line EGFR positive NSCLC showed significantly improved PFS with afatinib but OS was not significantly improved.

## Introduction

The advent of targeted therapy has had a dramatic effect on the treatment of cancer. Few treatment landscapes have shifted more in recent years than in metastatic non-small cell lung cancer (NSCLC). The identification of several oncogenic driver mutations has led to the development of targeted agents (1). The principal targets identified include rearrangements in the anaplastic lymphoma kinase gene and mutations of the epidermal growth factor receptor (EGFR) (1–4).

Epidermal growth factor receptor is a receptor that is part of the ErbB family (5, 6). This family of receptors includes four members; human epidermal growth factor 1 (HER1; EGFR, ErbB1), HER2 (Neu, ErbB2), HER3 (ErbB3), and HER4 (ErbB4) (5, 6). The physiological role of these receptor tyrosine kinases is to regulate cellular proliferation (5). Somatic EGFR mutations are present in around 50% of patients in Asia and in 10–15% of Caucasian patients with metastatic NSCLC with adenocarcinoma histology (7). Most of these mutations are caused by deletions on the exon 19 or L858R point mutations on exon 21 (8). EGFR-activating mutations lead to aberrant constitutive signaling by EGFR and its associated cell signaling pathways. As a consequence, proliferation often becomes completely dependent on EGFR activation in a phenomenon known as oncogene addiction. Because of this, inhibition of EGFR interrupts proliferation and induces apoptosis (9).

Epidermal growth factor receptor inhibition with oral tyrosine kinase inhibitors (TKIs) has shown proven clinical benefit in patients with NSCLC harboring activating EGFR mutations. The first-generation EGFR-TKIs gefitinib and erlotinib have demonstrated improved progression-free survival (PFS) and response rates but not overall survival (OS) in randomized phase III trials when compared with platinum-doublet chemotherapy (10–16).

## First-Generation EGFR TKIs: Gefitinib and Erlotinib

The first-generation EGFR-TKIs, gefitinib and erlotinib, bind reversibly to the kinase domain of the receptor. This leads to the inhibition of both mutant and, to a lesser extent, wild-type EGFR (17). In the early phase III trials of gefitinib conducted in Asia, IPASS, and First SIGNAL (Table 1) (10, 13), patients were not initially selected for their EGFR mutation status. Several subgroup analyses of these trials in addition to smaller subsequent trials, however, showed that the presence of EGFR-activating mutations was a strong predictor of clinical benefit with gefitinib when compared with platinum-doublet chemotherapy (10, 13, 18, 19). As a result, subsequent phase III trials of EGFR-TKIs included exclusively patients with activating EGFR mutations (11, 12, 14, 16). Two additional phase III trials, NEJ002 and WJTOG3405, also showed significant PFS advantages of first-line gefitinib when compared to chemotherapy, this time in a Japanese EGFR-mutant population (Table 1) (11, 12).

**Table 1:** **Randomized phase III trials comparing EGFR TKIs to standard platinum-based chemotherapy for first-line treatment of advanced *EGFR* mutation-positive NSCLC [adapted from Ref. (20)]**.

TKI	Reference	Study	Geography	Comparator	No. of pts^a^	RR (%)	Median PFS^b^ (months)	Difference in PFS, HR (95% CI); *p*-value	Median OS (months)	Difference in OS, HR (95% CI); *p*-value	Difference in OS—Del19 mutation, HR (95% CI); *p*-value
Gefitinib	(13, 21, 22)	IPASS	East Asia	Carboplatin + paclitaxel	261	71 vs. 47	9.5 vs. 6.3^d^	0.48 (0.36–0.64); *p* < 0.001	21.6 vs. 21.9	1.00 (0.76–1.33); *p* = n.s.	0.79 (0.54–1.15)^c^ *p* = n.s.
	(10)^c^	First-SIGNAL^c^	South Korea	Cisplatin + gemcitabine	42	85 vs. 38	8.0 vs. 6.3^d^	0.54 (0.27–1.1); *p* = n.s	27.2 vs. 25.6^b^	1.04 (0.50–2.18)^e^ *p* = n.s.	n/a
											*p* = n.s.
	(12)	WJTOG 3405^f^	Japan	Cisplatin + docetaxel	177	62 vs. 32	9.2 vs. 6.3^d^	0.49 (0.34–0.71); *p* < 0.0001	34.8 vs. 37.3^b^	1.25 (0.88–1.78)^e^ *p* = n.s.	n/a
											*p* = n.s.
	(11, 23)	NEJGSG 002^c^	Japan	Carboplatin + paclitaxel	230	74 vs. 31	10.8 vs. 5.4^g^	0.30 (0.22–0.41); *p* < 0.001	27.7 vs. 26.6	0.89 (0.63–1.24); *p* = n.s.	0.83 (0.52–1.34)^e^
											*p* = n.s.
Erlotinib	(16, 24)	OPTIMAL	China	Carboplatin + gemcitabine	154	83 vs. 36	13.1 vs. 4.6^d^	0.16 (0.10–0.26); *p* < 0.0001	22.7 vs. 28.9^b^	1.04 (0.69–1.58); *p* = n.s.	n/a
											*p* = n.s.
	(14)	EURTAC	France, Italy, Spain	Cisplatin or carboplatin^h^ + docetaxel or gemcitabine	173	58 vs. 15	9.7 vs. 5.2^g^	0.37 (0.25–0.54); *p* < 0.0001	19.3 vs. 19.5^b^	1.04 (0.65–1.68); *p* = n.s.	0.94 (0.57–1.54)^e^ *p* = n.s.
Afatinib	(21, 25)	LL3	Global	Cisplatin + pemetrexed	345	56 vs. 23	13.6 vs. 6.9^g^	0.47 (0.34–0.65); *p* = 0.001	31.6 vs. 28.2^b^	0.78 (0.58–1.06); *p* = n.s.	0.54 (0.36–0.79); *p* = 0.0015
	(21, 26)	LL6	China, South Korea	Cisplatin + gemcitabine	364	67 vs. 23	11.0 vs. 5.6^g^	0.28 (0.20–0.39); *p* < 0.0001	23.6 vs. 23.5^b^	0.83 (0.62–1.09); *p* = n.s.	0.64 (0.44–0.94); *p* = 0.023

*CI, confidence interval; EGFR, epidermal growth factor receptor; EURTAC, European tarceva vs. chemotherapy; First-SIGNAL, First-line single-agent iressa vs. gemcitabine and cisplatin trial in never-smokers with adenocarcinoma of the lung; HR, hazard ratio; IPASS, Iressa Pan-Asia study; n/a, not available; n.s., not significant; NEJGSG, North East Japan Gefitinib Study Group; LL3, LUX-Lung 3; LL6, LUX-Lung 6; NSCLC, non-small cell lung cancer; OS, overall survival; PFS, progression-free survival; RR, response rate; TKI, tyrosine kinase inhibitor; WJTOG, West Japan Thoracic Oncology Group*.

*^a^Number of patients enrolled with EGFR mutations*.

*^b^In patients with common activating mutations (*Del19* and/or *L858R*)*.

*^c^Patients with EGFR mutations were a subgroup of all enrollees*.

*^d^Based on investigator assessment*.

*^e^No p-value reported*.

*^f^Including patients with either postoperative recurrent or stage IIIb/IV NSCLC*.

*^g^Based on independent central review*.

*^h^Carboplatin plus docetaxel or gemcitabine was allowed for patients for whom cisplatin was contraindicated*.

The benefit of EGFR-TKIs was also demonstrated in a European population with advanced NSCLC and EGFR-activating mutations. The phase III EURTAC trial compared erlotinib with platinum-based chemotherapy. Erlotinib was associated with a significant benefit in PFS and was better tolerated than chemotherapy (Table 1) (14). The OPTIMAL trial also showed similar results with erlotinib in a Chinese population (16).

Gefitinib and erlotinib have also shown efficacy in second and third line treatment of NSCLC (2). Erlotinib may be an option in both EGFR mutated and wild-type patients. This is based on the results of NCIC BR21 placebo-controlled phase III trial in which patients were not selected for EGFR status. The trial demonstrated a PFS advantage with docetaxel (27). When compared with docetaxel, however, erlotinib did not appear to benefit patients with wild-type EGFR tumors in two phase III trials. In the TAILOR trial, PFS was significantly longer in wild-type EGFR NSCLC patients treated with second line docetaxel (28). In the DELTA trial, no PFS or OS improvement was shown in an EGFR-unselected population treated in the second or third line (29).

Unfortunately, NSCLC with EGFR-activating mutations treated with first-generation EGFR-TKIs inevitably develop resistances (30). Several resistance mechanisms have been described. The development of a T790M missense mutation in exon 20 is the most common of these and has been described in 50–60% of patients (31–33). This mutation causes steric hindrance, which obstructs binding of EGFR-TKIs to their target receptor (34). Other reported resistance mechanisms include alterations to the MET receptor (35–37) and amplification of HER2 (35–37) and HER3 (38).

## Afatinib

Afatinib irreversibly inhibits the tyrosine kinase activity of EGFR, HER2, and ErbB4 by forming covalent bonds to the receptors (39). Although ErbB3 lacks intrinsic kinase activity, it does form active heterodimers by interacting with ErbB family receptors and with HER2 in particular (40). Afatinib suppresses the activity of all four ErbB family members (39). Its irreversible inhibition is also more potent and prolonged than the reversible first-generation EGFR-TKIs (17, 39, 41).

## First-Line Afatinib in Patients with NSCLC and Activating EGFR Mutations: LUX-Lung 3 (LL3) and LUX-Lung 6 (LL6)

The largest randomized phase III trials in treatment-naive advanced NSCLC with EGFR-activating mutations were the LL3 and LL6 trials. The LL3 trial was a global trial, which recruited 345 patients while the LL6 trial recruited 364 patients in Asia (15, 21, 25). Patients were randomized (2:1) to afatinib (40 mg/day) or up to six cycles of platinum-doublet chemotherapy. LL3 used cisplatin and pemetrexed as a control group while LL6 used cisplatin and gemcitabine (42). The primary endpoint of these trials was PFS by prespecified independent central review. The trials also included comprehensive patient-reported outcomes (PROs) related to functional health status/quality of life (QoL) and lung cancer-related symptoms (Table 2) (15, 25, 43).

**Table 2:** **Patient-reported outcome assessments in first-line *EGFR* mutation-positive clinical trials vs. platinum-doublets [adapted from Ref. (20)]**.

Trial	Treatments	QoL assessments	Methodology	Outcomes
IPASS (13)	Gefitinib vs. carboplatin + paclitaxel	FACT-L and FACT-TOI	Randomization, week 1, every 3 weeks until day 127, once every 6 weeks from day 128 until disease progression, and when the study drug was discontinued	Significantly more patients in the gefitinib group than in the carboplatin + paclitaxel group had a clinically relevant improvement in QoL and by scores on the FACT-TOI. Rates of reduction in symptoms were similar
EURTAC (14)	Erlotinib vs. cisplatin + docetaxel or gemcitabine	Completion of the lung cancer symptom scale	Baseline, every 3 weeks, end of treatment visit, and every 3 months during follow-up	Insufficient data collected for any analysis to be done—due to low compliance
LL3 (25, 43)	Afatinib vs. cisplatin + pemetrexed	EORTC QLQ-C30, EORTC	Baseline, every 3 weeks until disease progression	Afatinib improved lung cancer-related symptoms and QoL and delay of deterioration of symptoms compared with chemotherapy
		QLQ-LC13		
LL6 (26)	Afatinib vs. gemcitabine + cisplatin	EORTC QLQ-C30, EORTC	Baseline, every 3 weeks until disease progression	Afatinib improved lung cancer-related symptoms of cough, dyspnea, and pain and global health status/QoL compared with chemotherapy
		QLQ-LC13		

*EGFR, epidermal growth factor receptor; EURTAC, European tarceva vs. chemotherapy; EORTC, QLQ European Organization for Research and Treatment of Cancer Quality of Life Questionnaire; FACT-L, Functional Assessment of Cancer Therapy—Lung; FACT-TOI, Functional Assessment of Cancer Therapy—Trial Outcome Index; IPASS, Iressa Pan-Asia study; LL3, LUX-Lung 3; LL6, LUX-Lung 6; QLQ-LC13, Quality of Life Questionnaire—Lung Cancer Module; QoL, quality of life*.

Both trials demonstrated a significant median PFS benefit with first-line afatinib [11.1 vs. 6.9 months; hazard ratio (HR) 0.58 *p* = 0.001 in LL3 and 11.0 vs. 5.6 months; HR 0.28; *p* = 0.0001 in LL6; Table 1] (15, 25). A preplanned analysis indicated that the PFS advantage was greater in patients with common EGFR mutations (Del19 and/or L858R). However, afatinib also showed activity in some patients with select uncommon EGFR-activating mutations. A pooled analysis of LL3, LL6, and the phase II LUX-Lung 2 (44) trials showed a median PFS of 10.7 months in 38 patients with uncommon mutations of EGFR (45). The pooled analysis also demonstrated particularly poor outcomes with afatinib in patients with exon 20 insertions (median PFS 2.7 months, *n* = 23).

Afatinib also showed clinical benefit in patients with brain metastases (46). A subgroup analysis of 35 patients in LL3 demonstrated a trend toward improved median PFS when compared to chemotherapy [11.1 vs. 5.4 months (HR 0.52 *p* = 0.13)]. For 10 patients with intracranial progression, median time to progression was 11.6 months with afatinib and 5.5 months with chemotherapy (46).

The median OS results of both trials did not show significant statistical differences between afatinib and chemotherapy. The LL3 trial had a median follow-up of 41 months. Median OS was 28.2 months in the afatinib arm and 28.2 months in the chemotherapy arm (HR 0.88, *p* = 0.39). In LL6, the median OS was 23.1 months for afatinib and 23.5 months for chemotherapy (HR 0.93, *p* = 0.61). However, in a preplanned analysis including only patients harboring Del19 mutations in both trials, a significant median OS advantage was shown in favor of afatinib (33.3 vs. 21.1 months; HR 0.54, *p* = 0.0015 in LL3 and 31.4 vs. 18.4 months; HR 0.64, *p* = 0.0229; Table 1) (21).

Both the LL3 and the LL6 trials integrated comprehensive PRO evaluation, including both the EORTC QLQLC12 and QLQ-C30 questionnaires, to determine the effect of afatinib on QoL (47). This differed from the past trials such as IPASS (which used Functional Assessment of Cancer Therapy indices) and EURTAC (analysis of PROs was not possible due to insufficient data). This showed that prespecified lung cancer-related symptoms, including cough, dyspnea, and pain were improved with afatinib. In addition, time to deterioration was longer with afatinib when compared to the chemotherapy arms. LL3 demonstrated statistically significant delayed time to deterioration and improved mean scores over time for cough and dyspnea (25, 43). Pain was not statistically different. Similar results were seen in LL6 with the addition that both time to deterioration and mean score over time were improved for pain. Overall, afatinib was associated with statistically significant improvements from baseline in global health status/QoL in both trials (26).

In comparison to platinum-based chemotherapy, afatinib was relatively well tolerated in both LL3 and LL6. Common grade 3 or higher treatment-related adverse events (AEs) of afatinib (LL3/LL6) included diarrhea (14/5%), rash and acne (16/15%), stomatitis and mucositis (9/5%), and paronychia (11/0%). There were more treatment discontinuations due to AEs in the chemotherapy arm than in the afatinib arm in both trials (12 vs. 8% in LL3 and 40 vs. 6% in LL6). No patient discontinued treatment due to diarrhea as a lone AE.

The relatively low rate of treatment discontinuations of afatinib in both trials may be due to effective symptom control and/or protocol defined dose reductions (25, 26). The trials recommended dose reductions in 10 mg decrements to a minimum dose of 20 mg for grade 3 AEs or grade 2 AEs lasting a prolonged length of time (25, 26). These reductions were shown to decrease excessive plasma concentrations of afatinib and, therefore, reduced toxicity without compromising efficacy. In fact, dose reduction was not associated with an inferior PFS (25).

## Afatinib in Patients with Relapsed/Refractory NSCLC: LUX-Lung 1 (LL1) and LUX-Lung 5 (LL5)

The phase IIb/III trial LL1 compared afatinib at a dose of 50 mg/day to placebo in 585 patients with stage IIIb/IV NSCLC. It included patients who had failed up to two lines of chemotherapy and had been exposed to at least 12 weeks of a first generation EGFR-TKI (gefitinib and/or erlotinib) (48, 49). Although a positive EGFR mutation status was not required, EGFR status was known for 141 patients and, of these, 68% were EGFR positive. Patients were randomly assigned to afatinib or placebo. Afatinib did not lead to a benefit in the primary endpoint of median OS. The median OS was 10.8 months for afatinib and 12.0 months for the placebo arm (HR 1.08, *p* = 0.74). Despite the absence of benefit in OS, an improvement in median PFS was seen with afatinib (3.3 vs. 1.1 months; HR 0.38, *p* < 0.0001) (49). The prolongation of PFS was also associated with an overall improvement in lung cancer-related symptoms and EORTC global health status (48).

Another phase III trial, LL5, included 202 EGFR mutation-positive patients with progressive disease on a prior EGFR-TKI (gefitinib, erlotinib, or afatinib) (46). Patients were randomly assigned to a combination of afatinib and paclitaxel or to investigator’s choice of chemotherapy without an EGFR-TKI. The trial achieved its primary endpoint of PFS. The median PFS was 5.6 months with afatinib and paclitaxel and 2.8 months with chemotherapy alone (HR 0.60, *p* = 0.003). The secondary endpoint of objective response rate (ORR) was also significantly improved (32.1 vs. 13.2%, *p* < 0.005), but median OS was not significantly different (12.2 vs. 12.2 months, HR 1.00, *p* = 0.994). The results of LL5 demonstrated prospective evidence of the benefit of maintaining EGFR blockade beyond disease progression in oncogene-addicted lung cancer.

## Comparing Reversible and Irreversible ErbB Family Blockade: LUX-Lung 7 (LL7)

Lux-Lung 7 was an open-label trial comparing first-line afatinib (40 mg/day) to gefitinib (250 mg/day) in 319 EGFR mutation-positive advanced NSCLC patients. This was an exploratory phase IIb trial. In the primary analysis, afatinib significantly improved the co-primary endpoints of PFS and time-to-treatment failure (TTF) when compared to gefitinib. At a median follow-up of 27.3 months, the median PFS was 11.0 months with afatinib and 10.9 months with gefitinib (HR 0.73, *p* = 0.017). TTF was 13.7 months with afatinib and 11.5 months with gefitinib (HR 0.73, *p* = 0.007). The key secondary endpoint of ORR was also significantly improved (*p* = 0.008). The treatment discontinuation rate was 6% in both arms (50). The OS data were recently updated with a median follow-up of 42.6 months. The median OS was 27.9 vs. 24.5 months with a non-significant trend in favor of afatinib (HR 0.86, *p* = 0.2580). Analysis by EGFR mutation subtype showed a median OS of 30.7 months for afatinib compared to 26.4 months for gefitinib (HR 0.83, *p* = 0.2841) in patients with exon 19 deletion. In patients with a L858R mutation, there was a median OS of 25.0 months for afatinib compared to 21.2 months for gefitinib (HR 0.91, *p* = 0.6585) (51). LL7 again demonstrated that dose reductions of afatinib reduced drug-related AEs without compromising efficacy. Overall, irreversible ErbB family blockade with afatinib provided improved clinical benefit over the reversible EGFR-TKI gefitinib for patients with EGFR mutation-positive NSCLC (50).

## Afatinib in Second-Line Treatment for NSCLC of Squamous Cell (SCC) Histology: LUX-Lung 8 (LL8)

Approximately 30% of NSCLC are of squamous histology (52). Platinum-doublet chemotherapy remains recommended first-line treatment for the majority of these patients. The phase III LL8 trial compared second-line afatinib (40 mg/day) and erlotinib (150 mg/day) in 795 patients with stage IIIb/IV SCC of the lung that were EGFR-TKI-naïve and had failed treatment after four or more cycles of platinum-based chemotherapy. The primary endpoint of PFS by independent radiological review was significantly improved with afatinib. The median PFS was 2.6 months with afatinib compared to 1.9 months with erlotinib (HR 0.81, *p* = 0.010). In addition, the secondary endpoint of OS was also significantly improved with afatinib (7.9 vs. 6.8 months; HR 0.81, *p* = 0.008). Furthermore, results for disease-control rate (50.5 vs. 39.5%, *p* = 0.002), ORR (5.5 vs. 2.8%, *p* = 0.055), and global health status/QoL (35.7 vs. 28.3%, *p* = 0.041) were all also in favor of afatinib (53). Overall, the benefit of EGFR-TKIs in squamous cell NSCLC has been limited. Immune-checkpoint inhibitors are now the preferred second-line option or even first-line option for patients with positive PD-L1 expression (54).

## Conclusion

The development of ErbB-family blockers has significantly improved patient outcomes for patients with metastatic NSCLC. This is particularly true in patients with EGFR-activating driver mutations where three EGFR-TKIs, gefitinib, erlotinib, and afatinib were shown to have significant survival advantage over first-line platinum-based chemotherapy. Afatinib, an irreversible ErbB family blocker, was designed to decrease resistance to reversible EGFR-TKIs and, therefore, prolong response in the first-line setting. Afatinib remains the only EGFR-TKI to have demonstrated a significant OS advantage in comparison to chemotherapy in patients with EGFR Del19 mutations. Furthermore, head-to-head data of LL7 trial demonstrated an improvement in PFS and PROs with afatinib regardless of mutation type. The results of afatinib in brain metastases have also been promising. There continues to be significant developments in the field of EGFR mutation-positive NSCLC, a third-generation of EGFR-TKIs is already seeking to improve outcomes, especially with osimertinib in patients resistant to EGFR-TKIs due to T790M mutations.

## Author Contributions

SM and VH contributed to the conception and design of the work, the drafting and revising of its content, and gave final approval of the version to be published. They agree to be accountable for all aspects of the work in ensuring that questions related to the accuracy or integrity of the work are appropriately investigated and resolved.

## Conflict of Interest Statement

VH: advisory board participation for Boehringer Ingelheim, AstraZeneca, Roche, Merck, Pfizer, Amgen, and Bristol Myers-Squibb. SM: nothing to declare.

## References

[1] Zer A Leighl N. Promising targets and current clinical trials in metastatic non-squamous NSCLC. Front Oncol (2014) 4:329. 10.3389/fonc.2014.00329 25505733 PMC4243502

[2] Network, N.C.C. Non-Small Cell Lung Cancer Version 5.2017 – March 16, 2017. (2017). Available from: https://www.nccn.org/professionals/physician_gls/pdf/nscl.pdf (accessed May 3, 2017).

[3] Solomon B Wilner KD Shaw AT Current status of targeted therapy for anaplastic lymphoma kinase-rearranged non-small cell lung cancer. Clin Pharmacol Ther (2014) 95(1):15–23. 10.1038/clpt.2013.200 24091716

[4] Yap TA Popat S. Toward precision medicine with next-generation EGFR inhibitors in non-small-cell lung cancer. Pharmgenomics Pers Med (2014) 7:285–95. 10.2147/PGPM.S55339 25278773 PMC4178554

[5] Roskoski R Jr. The ErbB/HER family of protein-tyrosine kinases and cancer. Pharmacol Res (2014) 79:34–74. 10.1016/j.phrs.2013.11.002 24269963

[6] Yarden Y Pines G. The ERBB network: at last, cancer therapy meets systems biology. Nat Rev Cancer (2012) 12(8):553–63. 10.1038/nrc3309 22785351

[7] Chan BA Hughes BG. Targeted therapy for non-small cell lung cancer: current standards and the promise of the future. Transl Lung Cancer Res (2015) 4(1):36–54. 10.3978/j.issn.2218-6751.2014.05.01 25806345 PMC4367711

[8] Reguart N Remon J. Common EGFR-mutated subgroups (Del19/L858R) in advanced non-small-cell lung cancer: chasing better outcomes with tyrosine kinase inhibitors. Future Oncol (2015) 11(8):1245–57. 10.2217/fon.15.15 25629371

[9] Engelman JA Janne PA. Mechanisms of acquired resistance to epidermal growth factor receptor tyrosine kinase inhibitors in non-small cell lung cancer. Clin Cancer Res (2008) 14(10):2895–9. 10.1158/1078-0432.CCR-07-2248 18483355

[10] Han JY Park K Kim SW Lee DH Kim HY Kim HT First-SIGNAL: first-line single-agent iressa versus gemcitabine and cisplatin trial in never-smokers with adenocarcinoma of the lung. J Clin Oncol (2012) 30(10):1122–8. 10.1200/JCO.2011.36.8456 22370314

[11] Maemondo M Inoue A Kobayashi K Sugawara S Oizumi S Isobe H Gefitinib or chemotherapy for non-small-cell lung cancer with mutated EGFR. N Engl J Med (2010) 362(25):2380–8. 10.1056/NEJMoa0909530 20573926

[12] Mitsudomi T Morita S Yatabe Y Negoro S Okamoto I Tsurutani J Gefitinib versus cisplatin plus docetaxel in patients with non-small-cell lung cancer harbouring mutations of the epidermal growth factor receptor (WJTOG3405): an open label, randomised phase 3 trial. Lancet Oncol (2010) 11(2):121–8. 10.1016/S1470-2045(09)70364-X 20022809

[13] Mok TS Wu YL Thongprasert S Yang CH Chu DT Saijo N Gefitinib or carboplatin-paclitaxel in pulmonary adenocarcinoma. N Engl J Med (2009) 361(10):947–57. 10.1056/NEJMoa0810699 19692680

[14] Rosell R Carcereny E Gervais R Vergnenegre A Massuti B Felip E Erlotinib versus standard chemotherapy as first-line treatment for European patients with advanced EGFR mutation-positive non-small-cell lung cancer (EURTAC): a multicentre, open-label, randomised phase 3 trial. Lancet Oncol (2012) 13(3):239–46. 10.1016/S1470-2045(11)70393-X 22285168

[15] Wu YL Zhou C Liam CK Wu G Liu X Zhong Z First-line erlotinib versus gemcitabine/cisplatin in patients with advanced EGFR mutation-positive non-small-cell lung cancer: analyses from the phase III, randomized, open-label, ENSURE study. Ann Oncol (2015) 26(9):1883–9. 10.1093/annonc/mdv270 26105600

[16] Zhou C Wu YL Chen G Feng J Liu XQ Wang C Erlotinib versus chemotherapy as first-line treatment for patients with advanced EGFR mutation-positive non-small-cell lung cancer (OPTIMAL, CTONG-0802): a multicentre, open-label, randomised, phase 3 study. Lancet Oncol (2011) 12(8):735–42. 10.1016/S1470-2045(11)70184-X 21783417

[17] Riely GJ Politi KA Miller VA Pao W. Update on epidermal growth factor receptor mutations in non-small cell lung cancer. Clin Cancer Res (2006) 12(24):7232–41. 10.1158/1078-0432.CCR-06-0658 17189394

[18] Jackman DM Miller VA Cioffredi LA Yeap BY Jänne PA Riely GJ Impact of epidermal growth factor receptor and KRAS mutations on clinical outcomes in previously untreated non-small cell lung cancer patients: results of an online tumor registry of clinical trials. Clin Cancer Res (2009) 15(16):5267–73. 10.1158/1078-0432.CCR-09-0888 19671843 PMC3219530

[19] Rosell R Moran T Queralt C Porta R Cardenal F Camps C Screening for epidermal growth factor receptor mutations in lung cancer. N Engl J Med (2009) 361(10):958–67. 10.1056/NEJMoa0904554 19692684

[20] Hirsh V. Next-generation covalent irreversible kinase inhibitors in NSCLC: focus on afatinib. BioDrugs (2015) 29(3):167–83. 10.1007/s40259-015-0130-9 26123538 PMC4488453

[21] Yang JC Wu YL Schuler M Sebastian M Popat S Yamamoto N Afatinib versus cisplatin-based chemotherapy for EGFR mutation-positive lung adenocarcinoma (LUX-Lung 3 and LUX-Lung 6): analysis of overall survival data from two randomised, phase 3 trials. Lancet Oncol (2015) 16(2):141–51. 10.1016/S1470-2045(14)71173-8 25589191

[22] Fukuoka M Wu YL Thongprasert S Sunpaweravong P Leong SS Sriuranpong V Biomarker analyses and final overall survival results from a phase III, randomized, open-label, first-line study of gefitinib versus carboplatin/paclitaxel in clinically selected patients with advanced non-small-cell lung cancer in Asia (IPASS). J Clin Oncol (2011) 29(21):2866–74. 10.1200/JCO.2010.33.4235 21670455

[23] Inoue A Kobayashi K Maemondo M Sugawara S Oizumi S Isobe H Updated overall survival results from a randomized phase III trial comparing gefitinib with carboplatin-paclitaxel for chemo-naive non-small cell lung cancer with sensitive EGFR gene mutations (NEJ002). Ann Oncol (2013) 24(1):54–9. 10.1093/annonc/mds214 22967997

[24] Zhou C Wu YL Chen G Feng J Liu XQ Wang C Final overall survival results from a randomised, phase III study of erlotinib versus chemotherapy as first-line treatment of EGFR mutation-positive advanced non-small-cell lung cancer (OPTIMAL, CTONG-0802). Ann Oncol (2015) 26(9):1877–83. 10.1093/annonc/mdv276 26141208

[25] Sequist LV Yang JC Yamamoto N O’Byrne K Hirsh V Mok T Phase III study of afatinib or cisplatin plus pemetrexed in patients with metastatic lung adenocarcinoma with EGFR mutations. J Clin Oncol (2013) 31(27):3327–34. 10.1200/JCO.2012.44.2806 23816960

[26] Wu YL Zhou C Hu CP Feng J Lu S Huang Y Afatinib versus cisplatin plus gemcitabine for first-line treatment of Asian patients with advanced non-small-cell lung cancer harbouring EGFR mutations (LUX-Lung 6): an open-label, randomised phase 3 trial. Lancet Oncol (2014) 15(2):213–22. 10.1016/S1470-2045(13)70604-1 24439929

[27] Shepherd FA Rodrigues Pereira J Ciuleanu T Tan EH Hirsh V Thongprasert S Erlotinib in previously treated non-small-cell lung cancer. N Engl J Med (2005) 353(2):123–32. 10.1056/NEJMoa050753 16014882

[28] Garassino MC Martelli O Broggini M Farina G Veronese S Rulli E Erlotinib versus docetaxel as second-line treatment of patients with advanced non-small-cell lung cancer and wild-type EGFR tumours (TAILOR): a randomised controlled trial. Lancet Oncol (2013) 14(10):981–8. 10.1016/S1470-2045(13)70310-3 23883922

[29] Kawaguchi T Ando M Asami K Okano Y Fukuda M Nakagawa H Randomized phase III trial of erlotinib versus docetaxel as second- or third-line therapy in patients with advanced non-small-cell lung cancer: docetaxel and erlotinib lung cancer trial (DELTA). J Clin Oncol (2014) 32(18):1902–8. 10.1200/JCO.2013.52.4694 24841974

[30] Cheng X Chen H. Tumor heterogeneity and resistance to EGFR-targeted therapy in advanced nonsmall cell lung cancer: challenges and perspectives. Onco Targets Ther (2014) 7:1689–704. 10.2147/OTT.S66502 25285017 PMC4181629

[31] Arcila ME Oxnard GR Nafa K Riely GJ Solomon SB Zakowski MF Rebiopsy of lung cancer patients with acquired resistance to EGFR inhibitors and enhanced detection of the T790M mutation using a locked nucleic acid-based assay. Clin Cancer Res (2011) 17(5):1169–80. 10.1158/1078-0432.CCR-10-2277 21248300 PMC3070951

[32] Pao W Miller VA Politi KA Riely GJ Somwar R Zakowski MF Acquired resistance of lung adenocarcinomas to gefitinib or erlotinib is associated with a second mutation in the EGFR kinase domain. PLoS Med (2005) 2(3):e73. 10.1371/journal.pmed.0020017 15737014 PMC549606

[33] Sequist LV Waltman BA Dias-Santagata D Digumarthy S Turke AB Fidias P Genotypic and histological evolution of lung cancers acquiring resistance to EGFR inhibitors. Sci Transl Med (2011) 3(75):75ra26. 10.1126/scitranslmed.3002003 21430269 PMC3132801

[34] Inukai M Toyooka S Ito S Asano H Ichihara S Soh J Presence of epidermal growth factor receptor gene T790M mutation as a minor clone in non-small cell lung cancer. Cancer Res (2006) 66(16):7854–8. 10.1158/0008-5472.CAN-06-1951 16912157

[35] Bean J Brennan C Shih JY Riely G Viale A Wang L MET amplification occurs with or without T790M mutations in EGFR mutant lung tumors with acquired resistance to gefitinib or erlotinib. Proc Natl Acad Sci U S A (2007) 104(52):20932–7. 10.1073/pnas.0710370104 18093943 PMC2409244

[36] Engelman JA Zejnullahu K Mitsudomi T Song Y Hyland C Park JO MET amplification leads to gefitinib resistance in lung cancer by activating ERBB3 signaling. Science (2007) 316(5827):1039–43. 10.1126/science.1141478 17463250

[37] Peled N Wynes MW Ikeda N Ohira T Yoshida K Qian J Insulin-like growth factor-1 receptor (IGF-1R) as a biomarker for resistance to the tyrosine kinase inhibitor gefitinib in non-small cell lung cancer. Cell Oncol (Dordr) (2013) 36(4):277–88. 10.1007/s13402-013-0133-9 23619944 PMC4186686

[38] Sergina NV Rausch M Wang D Blair J Hann B Shokat KM Escape from HER-family tyrosine kinase inhibitor therapy by the kinase-inactive HER3. Nature (2007) 445(7126):437–41. 10.1038/nature05474 17206155 PMC3025857

[39] Solca F Dahl G Zoephel A Bader G Sanderson M Klein C Target binding properties and cellular activity of afatinib (BIBW 2992), an irreversible ErbB family blocker. J Pharmacol Exp Ther (2012) 343(2):342–50. 10.1124/jpet.112.197756 22888144

[40] Aurisicchio L Marra E Luberto L Carlomosti F De Vitis C Noto A Novel anti-ErbB3 monoclonal antibodies show therapeutic efficacy in xenografted and spontaneous mouse tumors. J Cell Physiol (2012) 227(10):3381–8. 10.1002/jcp.24037 22213458

[41] Li D Ambrogio L Shimamura T Kubo S Takahashi M Chirieac LR BIBW2992, an irreversible EGFR/HER2 inhibitor highly effective in preclinical lung cancer models. Oncogene (2008) 27(34):4702–11. 10.1038/onc.2008.109 18408761 PMC2748240

[42] Langer CJ Epidermal growth factor receptor inhibition in mutation-positive non-small-cell lung cancer: is afatinib better or simply newer? J Clin Oncol (2013) 31(27):3303–6. 10.1200/JCO.2013.49.8782 23980079

[43] Yang JC Hirsh V Schuler M Yamamoto N O’Byrne KJ Mok TS Symptom control and quality of life in LUX-Lung 3: a phase III study of afatinib or cisplatin/pemetrexed in patients with advanced lung adenocarcinoma with EGFR mutations. J Clin Oncol (2013) 31(27):3342–50. 10.1200/JCO.2012.46.1764 23816967

[44] Yang JC Shih JY Su WC Hsia TC Tsai CM Ou SH Afatinib for patients with lung adenocarcinoma and epidermal growth factor receptor mutations (LUX-Lung 2): a phase 2 trial. Lancet Oncol (2012) 13(5):539–48. 10.1016/S1470-2045(12)70086-4 22452895

[45] Yang JC Sequist LV Geater SL Tsai CM Mok TS Schuler M Clinical activity of afatinib in patients with advanced non-small-cell lung cancer harbouring uncommon EGFR mutations: a combined post-hoc analysis of LUX-Lung 2, LUX-Lung 3, and LUX-Lung 6. Lancet Oncol (2015) 16(7):830–8. 10.1016/S1470-2045(15)00026-1 26051236

[46] Schuler M Yang JC Park K Kim JH Bennouna J Chen YM Afatinib beyond progression in patients with non-small-cell lung cancer following chemotherapy, erlotinib/gefitinib and afatinib: phase III randomized LUX-Lung 5 trial. Ann Oncol (2016) 27(3):417–23. 10.1093/annonc/mdv597 26646759 PMC4769992

[47] Hirsh V. Are the data on quality of life and patient reported outcomes from clinical trials of metastatic non-small-cell lung cancer important? World J Clin Oncol (2013) 4(4):82–4. 10.5306/wjco.v4.i4.82 24926427 PMC4053709

[48] Hirsh V Cadranel J Cong XJ Fairclough D Finnern HW Lorence RM Symptom and quality of life benefit of afatinib in advanced non-small-cell lung cancer patients previously treated with erlotinib or gefitinib: results of a randomized phase IIb/III trial (LUX-Lung 1). J Thorac Oncol (2013) 8(2):229–37. 10.1097/JTO.0b013e3182773fce 23328549

[49] Miller VA Hirsh V Cadranel J Chen YM Park K Kim SW Afatinib versus placebo for patients with advanced, metastatic non-small-cell lung cancer after failure of erlotinib, gefitinib, or both, and one or two lines of chemotherapy (LUX-Lung 1): a phase 2b/3 randomised trial. Lancet Oncol (2012) 13(5):528–38. 10.1016/S1470-2045(12)70087-6 22452896

[50] Park K Tan EH O’Byrne K Zhang L Boyer M Mok T Afatinib versus gefitinib as first-line treatment of patients with EGFR mutation-positive non-small-cell lung cancer (LUX-Lung 7): a phase 2B, open-label, randomised controlled trial. Lancet Oncol (2016) 17(5):577–89. 10.1016/S1470-2045(16)30033-X 27083334

[51] Paz-Ares L Tan EH Zhang L Hirsh V O’Byrne K Boyer M Afatinib versus Gefitinib in Patients with EGFR Mutation-Positive NSCLC: Overall Survival Data from the Phase IIB Trial LUX-Lung 7, in ESMO Meeting. Copenhagen, Denmark (2016). 10.1093/annonc/mdw611 PMC5391700 28426106

[52] Bryant A Cerfolio RJ. Differences in epidemiology, histology, and survival between cigarette smokers and never-smokers who develop non-small cell lung cancer. Chest (2007) 132(1):185–92. 10.1378/chest.07-0442 17573517

[53] Soria JC Felip E Cobo M Lu S Syrigos K Lee KH Afatinib versus erlotinib as second-line treatment of patients with advanced squamous cell carcinoma of the lung (LUX-Lung 8): an open-label randomised controlled phase 3 trial. Lancet Oncol (2015) 16(8):897–907. 10.1016/S1470-2045(15)00006-6 26156651

[54] Reck M Rodríguez-Abreu D Robinson AG Hui R Csőszi T Fülöp A Pembrolizumab versus chemotherapy for PD-L1-positive non-small-cell lung cancer. N Engl J Med (2016) 375(19):1823–33. 10.1056/NEJMoa1606774 27718847

